# Transepithelial Gingival Depigmentation Using a New Protocol with Q-Switched Nd:YAG: An In Vivo Observational Study

**DOI:** 10.3390/dj11010002

**Published:** 2022-12-21

**Authors:** Niccolò Giuseppe Armogida, Carlo Rengo, Mariangela Cernera, Flavia Iaculli, Gianrico Spagnuolo

**Affiliations:** Department of Neuroscience, Reproductive Sciences and Dentistry, University of Naples Federico II, 80126 Naples, Italy

**Keywords:** esthetic, gingival hyperpigmentation, laser, melanin, Nd:YAG laser

## Abstract

Gingival melanin hyperpigmentation is a para-physiological condition that may have a negative impact on smile esthetics. In the present study, the use of the Q-Switched Nd:YAG laser, according to a defined protocol, was proposed to treat Gingival Melanin Hyperpigmentation with a transepithelial approach. A total of 10 Patients with different grades of gingival hyperpigmentation were treated with Q-Switched Nd:YAG in one to four laser sessions without local anesthesia. The grade of depigmentation was evaluated by comparing Oral Pigmentation Index (OPI) and Melanin Pigmentation Index (MPI) at baseline and three weeks after the laser session. Additionally, oral discomfort rated by the Numeric Rating Scale (NRS) was recorded one, three, and five days after the procedure. Complete depigmentation was achieved in all cases. Patients reported no-little discomfort (NRS 0 to 3) during the laser session that lasted a maximum of five days. No major complications were reported, and no recurrences were observed at least after one year of follow-up. In addition, patients were available to be re-treated if necessary. These findings suggested that the Q-Switched Nd:YAG could be an effective and well-tolerated approach in the treatment of gingival melanin hyperpigmentation.

## 1. Introduction

In recent years, a perfect smile has increasingly become a main objective of patients’ esthetic requests. This is influenced by the shape and position of teeth, as well as the “pink aesthetic”. The natural color of gingiva is coral pink; however, it might change due to the thickness of the keratinized epithelium, degree of vascularization, and presence of pigments, as a result of the number and/or activity of melanocytes [1,2].

Gingival Melanin hyperpigmentation is a para-physiological condition and varies in different ethnic groups, sometimes causing a negative impact on smile esthetics [3].

Conventional treatments suggested in the literature for gingival depigmentation are gingivoplasty [4], abrasion [5], electrosurgery [6], and cryosurgery [7]. All these techniques are quite invasive, report some complications, require large compliance of patients, and may have the shortcoming of recurrence, even after only 33 days [8]. In addition, it should be considered that the definite mechanism of re-pigmentation has not yet been clarified [9]. 

In the last years, new technologies have been successfully applied in the dentistry field to obtain less invasive and more rapid treatments and improve patients’ compliance; in this view, several lasers with different parameters have been proposed [10,11,12,13,14]. Different lasers vary in wavelength and interaction with tissues and may be divided depending on two distinct approaches: the ablative (i.e., diode laser, Er,Cr:YSGG, Er:YAG, CO_2_) in which the epithelium is removed up to the pigmentation, and the non-ablative, in which epithelium is not removed [15].

To treat hyperpigmentation, an Nd:YAG laser—a solid-state laser that uses a yttrium-aluminum-garnet crystal doped at 1% with a semiconductor (neodymium) that gives the crystal particular physical properties—has been proposed in combination with Q-Switched technology, characterized by the release of a high amount of energy in nanosecond [16]. The administration of energy in such a short pulse causes a rapid thermal expansion of the target that is subsequently fragmented by the release of an acoustic wave leading to the mechanical destruction of neighboring tissue [17]. A laser pulse hits the pigment and destroys it into smaller fragments that will be phagocyted by macrophages and removed by the lymphatic system [18]. Nd:YAG laser is able to lightly penetrate into tissues, remaining in the transepithelial layers, without damaging the surface. In addition, it emits light with a wavelength of either 1064 nm or frequency-doubled 532 nm; the latter acts on red and brown pigments [17], and it is highly absorbed by melanosomes [19].

Nd:YAG laser was applied in a few studies treating gingival hyperpigmentation [9,15]; however, they did not include only melanin pigmentations but also smoking ones and reported a small sample with a short follow-up [9,15].

Therefore, the aim of the present study was to determine the efficacy of the Q-Switched Nd:YAG for the definitive treatment of gingival melanin hyperpigmentation.

## 2. Materials and Methods

### 2.1. Study Design and Participants 

The present work was an observational retrospective study carried out between January 2019 to January 2020 at the University of Naples Federico II, Department of Neuroscience, Reproductive Sciences and Dentistry. Since data collection for retrospective analysis was not considered a new clinical study, the same did not require any approval from the ethical committee of the University of Naples Federico II.

The study was conducted according to the Helsinki Declaration, and the methods conformed with the Strengthening the Reporting of Observational Studies in Epidemiology (STROBE) guidelines for observational studies. Patients enrolled in the present retrospective study were identified through clinical charts presenting gingival pigmentation caused by an accumulation of melanin and seeking treatment. The following criteria were used:

Inclusion criteria: (a) either gender, (b) aged 18 years or older, (c) diagnosis of physiological melanin gingival hyperpigmentation based on Meleti’s flowchart [20] with a score 1 or more according to the oral pigmentation index (OPI) [21].

Exclusion criteria: (a) patients with pigmentation affecting other areas (skin or of the oral mucosa), (b) presence of other gingival diseases such as leukoplakia, lichen planus, or other pigmented lesions (e.g., amalgama pigmentation, melanoma or other malignant pigmentation), (c) patients with a diagnosis of gingivitis, (d) patients with a diagnosis of periodontitis, (e) patients with systemic disease or condition that could affect tissue healing (e.g., autoimmune disease), (f) patients with a history of smoking, (g) patients with a previous mucogingival surgical treatment in the hyperpigmented region, (h) patients unwilling to receive laser therapy.

To define the grade of gingival pigmentation, two indexes were used: Oral Pigmentation Index (OPI) [21] and Melanin Pigmentation Index (MPI) [22]:

Oral Pigmentation Index (OPI) [21]:

- Score 0: no clinical pigmentation (pink-colored gingiva);
- Score 1: mild clinical pigmentation (mild light brown color);
- Score 2: moderate clinical pigmentation (medium brown or mixed pink and brown);
- Score 3: heavy clinical pigmentation (deep brown or bluish-black color).

Melanin Pigmentation Index (MPI) [22]:

- Score 0: no pigmentation;
- Score 1: solitary unit(s) of pigmentation in the papillary gingiva without extension between neighboring solitary units;
- Score 2: formation of continuous ribbon extending from neighboring solitary units.

### 2.2. Laser Treatment

At the first visit, patients underwent a comprehensive intra- and extra-oral examination, and data on demographic characteristics (age, gender), medical history, skin characteristics (Phototype evaluated using the Fitzpatrick Scale) [23] (Table 1), gingival phenotype (“Thin” and “Thick”) [24] and gingival pigmentation characteristics (OPI and MPI) [21,22] were recorded.

Pictures of gums were taken at first visit by using a Nikon D7500 with a Nikon AF-S 105 mm f/2.8 G ED VR Micro lens. Pictures were also taken immediately after the laser session, after 21 days, and every 21 days after the last treatment. In all cases, the same operator (NGA) used a Q-Switched Nd:YAG laser (Synchro QS4 Deka, M.E.L.A s.r.l., Calenzano (FI), Italy) with noncontact technique with a distance of 1 cm from the gums. The laser was set to release light energy at a wavelength of 532 nm.

The laser has been held perpendicular to the skin with the lesion at its focal point. The Q-Switched Nd:YAG laser was settled with the following operating parameters: fluence 2.3 J/cm^2^; frequency 2 Hz and spot size 2.5 mm were set. 

Laser pulse was shot on all pigmented areas until the area was brisk whitening, and a characteristic appearance called “frosting” was achieved. The frosting is normally observed during the tattoo removal with the Q-switched Nd:YAG laser, and it happens because of release of gas bubbles in stratum corneum [25]. In all cases, no local anesthesia was required. The patients and the operator wore protective eye coverings.

No additional substance or material was used to encourage the healing process. The patient was suggested to avoid acidic foods or beverages during the first week.

### 2.3. Outcomes Assessment 

The primary outcome was to obtain complete depigmentation of the gums. 

The depigmentation was measured 3 weeks after the laser session by two independent clinicians. The preoperative and postoperative areas of each lesion were compared, and the gingival appearance, including the homogeneity of the color of the gingiva, was evaluated using the OPI [21] and MPI [22] score: if score was >0 of both the Index, additional laser sessions were conducted until the complete depigmentation was obtained.

As secondary outcome, the pain/oral discomfort during the first laser session and after 1, 3, and 5 days were evaluated. All patients were asked to rate their pain/discomfort by using the Numeric Rating Scale (NRS-11). This is a validated tool for assessing pain intensity whose scale range from 0 to 10 (0 = no oral symptoms and 10 = the worst imaginable discomfort), that reported better patients’ compliance than traditional Visual Analogue Scale (VAS) [26]. Patient perceptions regarding the possibility of repeating the treatment, if necessary, were asked at 12 months follow-up, using dichotomic parameters as answer (NO—YES).

Other outcomes of interest were the presence of postoperative complications and relapses.

Treated subjects were followed up at 6 and 12 months post-treatment. 

## 3. Results

The study consisted of 10 patients, 3 (30%) males and 7 (70%) females, with a mean age of 41.5 years (range 21–72 years). Patients’ Phototypes varied from class III to VI. Gingival Phenotype was evaluated as “Thick” in 9 patients (90%) and “Thin” in 1 patient (10%).

All participants had at least 12 months of follow-up. 

Characteristics of all patients are reported in Table 2. 

The effectiveness of the depigmentation protocol was examined by comparing the OPI and MPI scores at baseline and at three weeks of follow-up. Five patients (50%) had the necessity to receive more than one laser session, specifically: one patient (10%) needed four laser sessions, three patients (30%) needed three laser sessions, and one patient (10%) needed two laser sessions. When the score of OPI was 1, only one laser session was needed to have complete depigmentation. However, in three patients (30%) with an OPI of 2, just one laser session was applied to reach complete depigmentation. In six patients with MPI of 2, the score was reduced from the first session, demonstrating that with one laser session, the continuous hyperpigmented ribbon (MPI = 2) became solitary units (MPI = 1) or completely disappeared (MPI = 0). However, in patient number 5, to obtain a reduction of MPI from 2 to 1, two sessions were needed. In all evaluated cases, pigmentations were completely removed after 12 months of follow-up (Table 3). 

**Figure 1:**
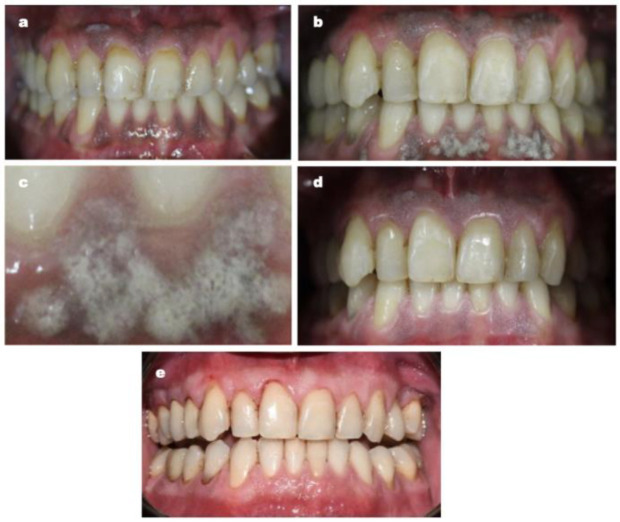
Patient no. 3: Preoperative optical findings (**a**); optical findings just after the laser application (**b**); frosting at higher magnification (**c**); optical findings immediately before the third laser application (**d**); 2 years after the treatment (**e**).

The proposed protocol at baseline, immediately after laser session and during the follow-up, is reported in Figure 1.

In patients receiving more than one laser session, the mean of the NRS evaluated at baseline and one, three, and five days after was calculated between the single session. Patients rated their oral pain/discomfort during the laser session between 0 and 3 according to the NRS scale, with a mean of 1.6. The day after the treatment, six patients had a slight discomfort (NRS from 2 to 4); the discomfort continued three days post-therapy in only four subjects (NRS from 1 to 3) (Table 4). 

**Figure 2:**
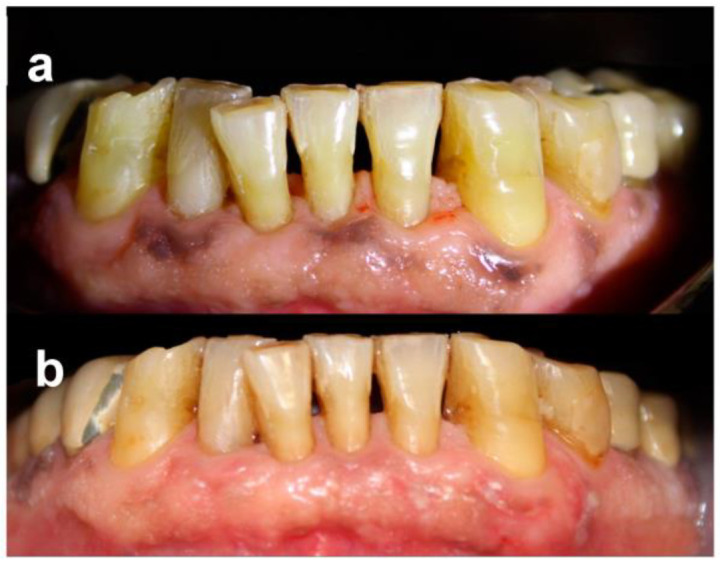
Patient no. 1: Preoperative clinical aspect (**a**) and follow-up 1 year after laser application (one session) (**b**).

**Figure 3:**
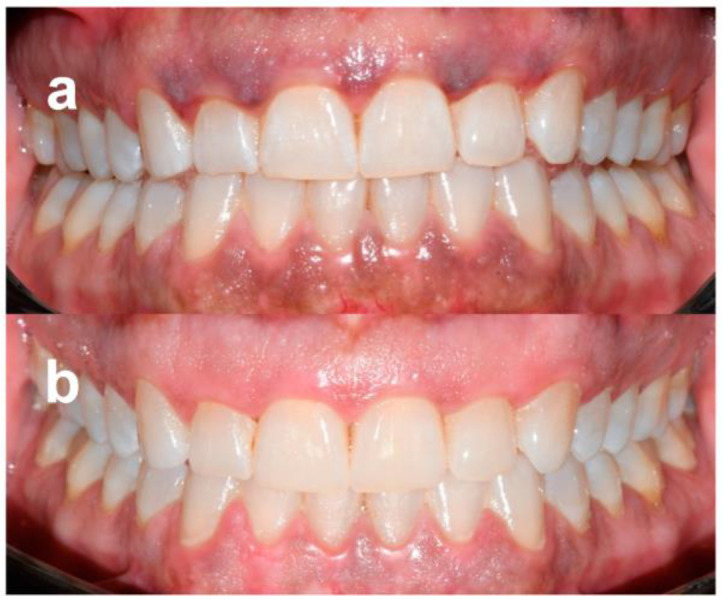
Patient no. 6: Preoperative clinical aspect (**a**) and follow-up 1 year after laser application (two sessions) (**b**).

Patients were followed-up regularly, and no recurrences were observed over a minimum follow-up of 12 months (Figure 2 and Figure 3). At the same time point, all patients were available to repeat the treatment if necessary (YES) (Table 4).

## 4. Discussion

Melanin gingival hyperpigmentation represents an aesthetic issue for many subjects, mainly if the hyperpigmentation is visible [27], and it frequently requires treatment. The gold standard for gingival depigmentation is still the blade scalped technique (gengivoplasty), although it is invasive and patients might have post-treatment discomfort [11,28]. Therefore, to avoid gingivoplasty-related complications, the use of a laser to obtain gingival depigmentation has recently been proposed [9,14,15,29,30].

The results of this study demonstrated that the Q-Swithced Nd:YAG protocols might be successfully applied for the treatment of gingival hyperpigmentation; indeed, in all treated patients, complete depigmentation was achieved with one to four laser sessions. Although, in some studies, different lasers were used only in one session [11,14,31]. A long time was required to obtain complete healing. In addition, this minimally invasive approach does not need any kind of local anesthesia, which is often necessary when the technique is ablative and involves the removal of gingival tissue [11,14,27,29,31,32].

Altayeb et al. [30] used two laser wavelengths (diode 940 nm and Er,Cr:YSGG 2780 nm) to treat gingival hyperpigmentation in an ablative way. Even though Er,Cr:YSGG reported faster treatment without anesthesia and less discomfort, a high percentage of patients showed postoperative bleeding that stopped in a maximum of seven days. In addition, the same study [30] evaluated intra-operatory and postoperative bleeding during therapy with both lasers. Bleeding was absent when the diode laser was applied; however, it occurred immediately after the procedure and stopped after three days in 57% of cases and seven days in 43% of cases. On the contrary, Er,Cr:YSGG laser demonstrated bleeding during the procedure and stopped in 13% of cases after one day, in 70% of cases after three days, and in 17% of cases, after seven days.

Moeintaghavi et al. [29] reported mild bleeding during the ablative procedure in 8% of treated cases using a diode laser with a pulsed mode, in 8% of cases using a diode laser with a continuous mode, and in 17% of cases using a CO_2_ laser. Accordingly, Harb et al. [31] had mild bleeding during the procedure with both diode and erbium laser, obtaining the complete healing of the injury in one month.

Riberio et al. [11] proposed the use of Nd:YAG with an ablative technique that had the advantage of reducing intra-operatory and post-operatory bleeding; however, it induced slower healing than the proposed technique with the persistence of slight discomfort during the week after. In this light, regardless of the ablative techniques, there was always intra-operatory and post-operatory bleeding [11,14,27,29,30,31] that may cause discomfort to patients. On the other hand, the technique proposed in the present study did not produce tissue removal, subsequent bleeding, and less discomfort in comparison with other laser treatments [11,29,30,31,32]. Er,Cr:YSGG depigmentation was demonstrated to be slightly painful, and in some cases, the pain persisted for more than a week [30]. Interestingly, our procedure had a similar or sometimes lower grade of pain that persisted for less than five days.

Regarding recurrences of pigmentation, a limited incidence was reported by Altayeb et al. [30] 1 month postoperatively using Er,Cr:YSGG, although more than 60% of treated patients had recurrences after one year. Ribero et al. [11] demonstrated a slight recurrence of pigmentation in almost 50% of patients after six months of follow-up. Accordingly, using an Er:YAG laser, Rosa et al. [32] observed a small recurrence in one of the five treated cases during a three-month clinical observation. On the contrary, the present study reported no cases of recurrence within at least one year of follow-up. This was probably due to the effect of Q-Switched Nd:YAG laser pulse that might be absorbed by the pigment-containing cells (such as the melanophages or melanocytes), reducing or even eliminating their activity. Moreover, acting directly on the melanosomes, clinicians are allowed to apply the treatment on the gingival margin and interdental papilla without having the risk of a gingival recession, which, in turn, represents the main drawback of ablative techniques [29].

Evaluation of patients’ perceptions of the discomfort felt during and after the procedure was a relevant parameter evaluated in the present study, considering the central role played by the subjects’ appreciation [33]. Indeed, all patients (100%) were willing to be re-treated if necessary. Altayeb et al. [30] reported the same parameter with almost 90% of consensus, probably because of the use of ablative technique, bleeding occurrence, and slower healing by secondary intention.

To the best of our knowledge, this is the first study evaluating pain and patients’ acceptance during hyperpigmentation removal treatment by means of a non-ablative technique. In this regard, further studies should also be focused on the relevance of patients’ acceptance and not only on the technical aspects of treatment.

## 5. Conclusions

Within the limitation of the present study and the limited sample size, the application of Q-Switched Nd:YAG laser in the treatment of gingival melanin hyperpigmentation might be a promising protocol demonstrating excellent esthetic outcomes with slight postoperative discomfort and no related complications. Nevertheless, further research and clinical trials comparing different procedures and with greater samples should be carried out in order to elucidate which is the most reliable and accepted treatment option.

## Figures and Tables

**Table 1:** Fitzpatrick Skin phototype classification.

	Fitzpatrick Classification
I	Pale white skin
II	Fair skin
III	Darker white to light brown skin
IV	Olive, light to moderate brown skin
V	Brown skin
VI	Dark brown or black skin

**Table 2:** Summary of patients’ demographics and clinical characteristics.

Patients	Sex	Age	Skin Type	Gingival Phenotype	OPI ^1^ at Baseline	MPI ^2^ at Baseline
1.	M	72	III	Thick	2	1
2.	F	28	III	Thick	1	1
3.	M	32	IV	Thick	2	2
4.	F	21	IV	Thin	2	2
5.	F	53	VI	Thick	3	2
6.	F	40	III	Thick	2	2
7.	F	49	III	Thick	1	2
8.	M	57	III	Thick	2	1
9.	F	36	IV	Thick	2	2
10.	F	27	V	Thick	2	2

^1^ OPI: Oral Pigmentation Index [21]. ^2^ MPI: Melanin Pigmentation Index [22].

**Table 3:** Summary of clinical outcomes.

	Patient 1	Patient 2	Patient 3	Patient 4	Patient 5	Patient 6	Patient 7	Patient 8	Patient 9	Patient 10
Laser session	1	1	3	1	4	2	1	1	2	3
Gingival pigmentation										
Baseline	OPI = 2 MPI = 1	OPI = 1 MPI = 1	OPI = 2 MPI = 2	OPI = 2 MPI = 2	OPI = 3 MPI = 2	OPI = 2 MPI = 2	OPI = 1 MPI = 2	OPI = 2 MPI = 1	OPI = 2 MPI = 2	OPI = 2 MPI = 2
1 session	OPI = 0 MPI = 0	OPI = 0 MPI = 0	OPI = 2 MPI = 1	OPI = 0 MPI = 0	OPI = 2 MPI = 2	OPI = 1 MPI = 1	OPI = 0 MPI = 0	OPI = 0 MPI = 0	OPI = 1 MPI = 1	OPI = 2 MPI =1
2 session	/	/	OPI = 1 MPI = 1	/	OPI = 2 MPI = 1	OPI = 0 MPI = 0	/	/	OPI = 0 MPI = 0	OPI = 1 MPI =1
3 session	/	/	OPI = 0 MPI = 0	/	OPI = 1 MPI = 1	/	/	/	/	OPI = 0 MPI = 0
4 session	/	/	/	/	OPI = 0 MPI = 0	/	/	/	/	/
Recurrence at 1 year of follow up	NO	NO	NO	NO	NO	NO	NO	NO	NO	NO

OPI: Oral Pigmentation Index [21]. MPI: Melanin Pigmentation Index [22].

**Table 4:** Summary of patients’ response outcomes.

Time	Patient 1	Patient 2	Patient 3	Patient 4	Patient 5	Patient 6	Patient 7	Patient 8	Patient 9	Patient 10
Pain (NRS ^1^)										
baseline	0	1	2	3	3	2	0	1	2	2
1 day	0	0	2	3	4	0	0	0	2	3
3 day	0	0	0	1	3	0	0	0	2	2
5 day	0	0	0	0	0	0	0	0	0	0
Discomfort at 12 months										
Would you repeat treatment if necessary?	YES	YES	YES	YES	YES	YES	YES	YES	YES	YES

^1^ NRS: Numeric Rating Score [26].

## Data Availability

Not applicable.
